# A Review of *β*‐Lactamase Inhibitors in Clinical Use and Development: Mechanisms, Spectrum, and Therapeutic Applications

**DOI:** 10.1155/ijm/4479031

**Published:** 2026-05-21

**Authors:** Mohammad Javad Roustaye Gourabi, Anita Nikoo, Bita Khanbabaei, Masoud Kargar, Amirhossein Fayyazi, Seyyed Mohammad Javad Mousavi, Amirhossein Aghdaee, Ali Hashemi, Javad Yasbolaghi Sharahi

**Affiliations:** ^1^ Department of Microbiology, School of Medicine, Shahid Beheshti University of Medical Sciences, Tehran, Iran, sbmu.ac.ir; ^2^ Department of Medical Parasitology and Entomology, Tarbiat Modares University, Tehran, Iran, modares.ac.ir; ^3^ Department of Biological Sciences, Roudehen Center, Islamic Azad University, Roudehen, Iran, azad.ac.ir; ^4^ Thalassemia and Hemoglobinopathy Research Center, Health Research Institute, Ahvaz Jundishapur University of Medical Sciences, Ahvaz, Iran, ajums.ac.ir; ^5^ Department of Pathobiology, School of Public Health, Tehran University of Medical Sciences, Tehran, Iran, tums.ac.ir; ^6^ Infectious Diseases and Tropical Medicine Research Center, Shahid Beheshti University of Medical Sciences, Tehran, Iran, sbmu.ac.ir; ^7^ Student Research Committee, Department of Microbiology, School of Medicine, Shahid Beheshti University of Medical Sciences, Tehran, Iran, sbmu.ac.ir

**Keywords:** *β*-lactam antibiotic, *β*-lactamase, *β*-lactamase inhibitor, antibiotic resistance, Gram-negative bacteria, metallo *β*-lactamase, multidrug resistance, serine *β*-lactamase

## Abstract

*β*‐Lactam antibiotics have long served as a cornerstone for treating bacterial infections. However, their widespread and often indiscriminate use has fueled the emergence of multidrug‐resistant Gram‐negative pathogens, primarily through the production and dissemination of *β*‐lactamase enzymes. The proliferation of these enzymes represents a critical threat to global public health, compounding therapeutic challenges and escalating healthcare costs. As the efficacy of conventional treatments diminishes, reliance on last‐resort agents like carbapenems and colistin has increased, in turn accelerating resistance to these precious final‐line options. To address this escalating crisis, the strategic combination of *β*‐lactam antibiotics with *β*‐lactamase inhibitors (BLIs) has emerged as a vital therapeutic approach, restoring the activity of *β*‐lactams by inactivating the resistance enzymes. Since the introduction of clavulanic acid, significant progress has been made in expanding the BLI arsenal. This review provides a comprehensive analysis of the current landscape of novel BLIs that have been approved or are in advanced clinical development, delving into their distinct mechanisms of action, spectra of activity against Ambler molecular classes (A, B, C, and D), and evolving clinical applications. While early inhibitors primarily targeted serine‐*β*‐lactamases (SBLs), recent advancements have introduced agents with expanded profiles, including those effective against some Class D carbapenemases. A paramount, ongoing challenge remains the development of effective inhibitors against metallo‐*β*‐lactamases (MBLs), though promising candidates like taniborbactam and xeruborbactam are now in clinical development. This review synthesizes the current knowledge on these innovative inhibitors, from recently approved combinations like ceftazidime/avibactam and cefepime/enmetazobactam to those in advanced clinical trials, and critically examines the associated therapeutic challenges, including the emergence of resistance. By integrating mechanistic insights with clinical perspectives, this article is aimed at informing the ongoing battle against antimicrobial resistance and guiding the future development of life‐saving therapeutic strategies.

## 1. Introduction

*β*‐Lactam antibiotics, including penicillins, cephalosporins, monobactams, and carbapenems, have served as the cornerstone of antibacterial therapy for decades. However, their widespread and often indiscriminate use has catalyzed the alarming emergence of multidrug‐resistant (MDR) superbugs, progressively diminishing the efficacy of these life‐saving agents [1, 2]. The genesis of this resistance traces back to 1940, when Abraham and Chain first documented bacterial enzymatic inactivation of penicillin, a phenomenon later attributed to *β*‐lactamases [1, 2]. Today, these enzymes have evolved into a diverse and sophisticated family, produced by both Gram‐positive and Gram‐negative pathogens, representing a paramount challenge in modern infectious disease management.

The persistence of *β*‐lactam resistance is largely driven by the bacterial capacity to overproduce and mutate *β*‐lactamases, which hydrolyze the critical *β*‐lactam ring, thereby inactivating the antibiotic [3]. To systematically classify these enzymes, two primary schemes are utilized: the Ambler molecular classification (Classes A, B, C, and D) and the functional classification by Bush [4]. Classes A, C, and D are serine *β*‐lactamases (SBLs) that employ an active‐site serine residue for catalysis. In contrast, Class B consists of MBLs, which are zinc‐dependent and represent a formidable resistance mechanism due to their broad substrate profile and intrinsic resistance to many conventional inhibitors [4].

The clinical ramifications of *β*‐lactamase‐mediated resistance are starkly evident in the global spread of carbapenem‐resistant *Enterobacterales* (CRE), featuring pathogens producing *Klebsiella pneumoniae* carbapenemase (*bla*_KPC_, Class A), New Delhi metallo‐*β*‐lactamase (*bla*_NDM_, Class B), and oxacillinase‐48 (*bla*_OXA-48_, Class D) [5–7]. These enzymes not only hydrolyze last‐resort carbapenems but are also frequently coproduced with other resistance determinants, leaving few therapeutic options.

**Figure 1:**
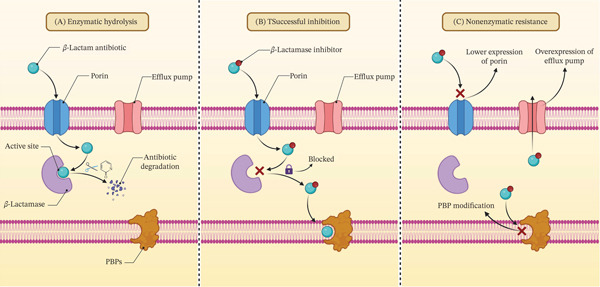
Schematic representation of bacterial resistance patterns: (A) Enzymatic hydrolysis‐mediated resistance. Bacterial resistance is achieved via the production of *β*‐lactamase enzymes, which hydrolyze and inactivate the antibiotic before it can bind to its target PBP. (B) Restoration of activity via *β*‐lactamase inhibition. Coadministration of a BLI inactivates the *β*‐lactamase enzyme. This inhibition allows the *β*‐lactam antibiotic to reach and bind its PBP target unimpeded, thereby restoring its bactericidal efficacy. (C) Nonenzymatic resistance mechanisms. Resistance can occur via pathways independent of enzymatic degradation: (1) downregulation of outer membrane porins, limiting antibiotic influx; (2) overexpression of efflux pumps, enhancing antibiotic expulsion; and (3) modification of the PBP target (e.g., acquisition of a low‐affinity PBP), which reduces antibiotic binding and efficacy.

To counter this crisis, BLIs are coadministered with *β*‐lactam antibiotics to restore their efficacy, as illustrated in Figure 1, which contrasts scenarios of inhibitor failure and success. Pioneering inhibitors like clavulanic acid, sulbactam (SUL), and tazobactam (TAZ) primarily target Class A enzymes but offer limited utility against Classes B, C, and D *β*‐lactamases [3].

**Figure 2:**
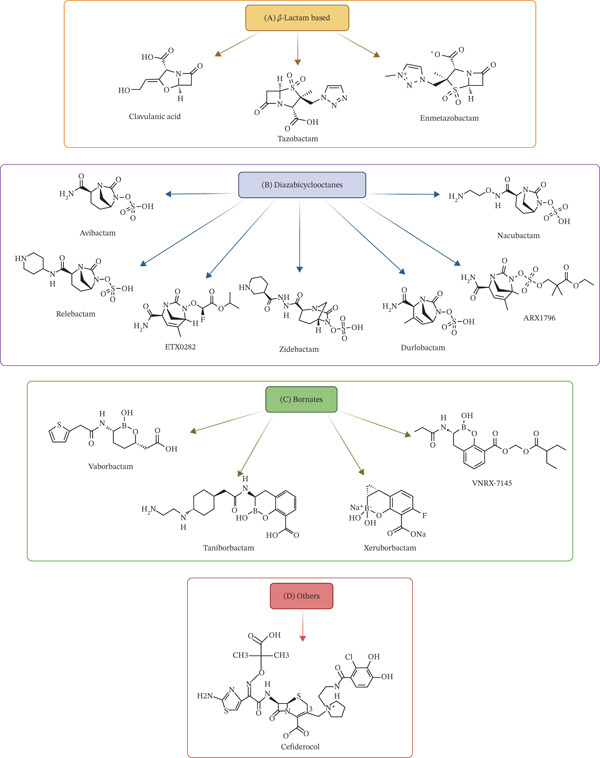
Chemical structure of *β*‐lactamase inhibitors. (A) *β*‐Lactam based; (B) diazabicyclooctanes; (C) bornates; and (D) others.

Recent medicinal chemistry advances have expanded the BLI arsenal, introducing novel agents such as avibactam (AVI) (a diazabicyclooctane) and vaborbactam (VAB) (a boronic acid derivative), which exhibit broader spectra encompassing some Classes C and D enzymes [3]. Despite this progress, the development of effective MBL inhibitors remains a critical unmet need, fueling intensive research into innovative chemical scaffolds, several of which are structurally depicted in Figure 2. This review delivers a comprehensive and up‐to‐date analysis of novel BLIs, emphasizing their mechanisms of action, clinical applications, and the therapeutic challenges they face. By integrating structural insights with clinical perspectives, we aim to evaluate the current landscape of inhibitors in clinical use and advanced development and provide a roadmap for future therapeutic development in this vital area. This review is organized according to the core chemical class and mechanism of action of the inhibitors. We begin with an overview of inhibition mechanisms, followed by detailed discussions of penicillanic acid sulfone derivatives, diazabicyclooctanes (DBOs), boronate‐based inhibitors, and agents with novel mechanisms. We also discuss the unique siderophore cephalosporin cefiderocol. Each section evaluates the inhibitors′ spectrum, clinical applications, and associated challenges. The article concludes with future perspectives on overcoming resistance.

## 2. Mechanisms of *β*‐Lactamase Inhibition and Inhibitor Design Strategies

The efficacy of BLIs hinges on their ability to outcompete the *β*‐lactam antibiotic for binding to the enzyme′s active site, thereby preventing hydrolysis and allowing the antibiotic to reach its target, the penicillin‐binding proteins (PBPs). The inhibition strategy and chemical design are fundamentally dictated by the class of *β*‐lactamase [8].

For SBLs, which utilize an active‐site serine nucleophile for catalysis, two primary inhibition mechanisms have been successfully employed. The first‐generation inhibitors, such as clavulanic acid and the penicillanic acid sulfones (e.g., TAZ and SUL), act as suicide substrates or mechanism‐based inactivators. They form an initial acyl–enzyme complex analogous to a *β*‐lactam substrate, but this intermediate undergoes further rearrangement (e.g., ring opening and tautomerization), leading to a permanently inactivated, covalently modified enzyme. The second, more recent strategy employs non–*β*‐lactam scaffolds that form reversible covalent complexes. The DBOs, such as AVI, function via carbamylation of the catalytic serine, creating a stable yet hydrolyzable bond that allows for potent inhibition without permanent enzyme destruction. Similarly, boronate‐based inhibitors (e.g., VAB) mimic the tetrahedral transition state of the hydrolysis reaction, forming reversible complexes with exceptional affinity, particularly against enzymes like *bla*_KPC_ [4, 9].

Inhibiting MBLs presents a distinct challenge, as these zinc‐dependent enzymes do not possess a catalytic serine. Effective MBL inhibitors often act as zinc chelators or transition‐state analogs that displace the activating water molecule. The development of broad‐spectrum inhibitors capable of inhibiting both SBLs and MBLs (e.g., taniborbactam [TAN] and xeruborbactam [XER]) represents a pinnacle of rational design, overcoming a major historical therapeutic gap [10].

Beyond pure enzyme inhibition, a novel design paradigm incorporates intrinsic antibacterial activity into the BLI scaffold. Some advanced inhibitors (e.g., zidebactam [ZID], nacubactam [NAC], and durlobactam [DUR]) not only inhibit *β*‐lactamases but also bind directly to PBP2. This dual‐action mechanism creates a powerful synergy with the partner *β*‐lactam (which typically targets PBP3), enhancing bacterial killing and potentially raising the barrier to resistance [11].

Finally, pharmacokinetic optimization, particularly for achieving oral bioavailability, has become a critical design goal. This has led to the development of ester prodrugs (e.g., ARX‐1796, ETX0282, and ledaborbactam etzadroxil) designed to mask polar groups, facilitating intestinal absorption before conversion to the active inhibitor in the systemic circulation [12].

The following sections review the major classes of BLIs that have reached clinical use or advanced development, organized according to these core chemical scaffolds and strategic design principles.

## 3. Penicillanic Acid Sulfone‐Based Inhibitors

The penicillanic acid sulfone scaffold represents the first generation of synthetic BLIs, developed through rational modification of the penicillin core. Characterized by a *β*‐lactam ring fused to a sulfone‐containing thiazolidine ring, these inhibitors act as irreversible, mechanism‐based inactivators of SBLs. While their spectrum is primarily focused on Class A enzymes, strategic optimizations and novel partnerships have sustained their clinical utility against specific resistance threats [13].

### 3.1. Clavulanic Acid: The Pioneering Inhibitor

#### 3.1.1. Discovery, Core Structure, and Pioneering Mechanism

Clavulanic acid, isolated from *Streptomyces clavuligerus*, was the first naturally occurring *β*‐lactamase inhibitor discovered and remains the archetype of its class. Its core structure, a clavam nucleus featuring a *β*‐lactam ring fused to an oxazolidine ring, differs fundamentally from penicillins and provided the first blueprint for a nonantibiotic *β*‐lactam molecule designed to inactivate resistance enzymes (Figure 2) [14, 15].

Its revolutionary mechanism of action is that of an irreversible suicide inhibitor. Clavulanic acid acts as a substrate analog for SBLs, primarily Ambler Class A enzymes such as *bla*_TEM_ and *bla*_SHV_ (Table 1). It is initially recognized and acylated by the catalytic serine of the enzyme. However, instead of undergoing hydrolysis, the resulting complex undergoes a series of irreversible rearrangements within the active site, leading to the permanent, covalent inactivation of the *β*‐lactamase. This process effectively disarms a key bacterial defense, allowing a coadministered *β*‐lactam antibiotic (most notably amoxicillin) to reach its PBP targets unimpeded [15, 16].

**Table 1:** Clinically approved *β*‐lactam/*β*‐lactamase inhibitor combinations.

Inhibitor	Partner *β*‐lactam	Combination (brand name)	Inhibition spectrum				Intrinsic PBP inhibition (by BLI alone)	Clinical status and year	Key approved indications	Key limitations
			A	B	C	D				
**Clavulanic acid**	Amoxicillin	Augmentin	+	—	—	—	None	Approved (1984)	RTIs, UTIs, skin/soft tissue infections	Narrow spectrum (Class A only); induces AmpC.
**Tazobactam**	Piperacillin	Zosyn/Tazocin	±	—	±	—	None	Approved (1993)	cIAI, cUTI, skin infections, CAP	Weak versus AmpC; no MBL or carbapenemase activity.
**Tazobactam**	Ceftolozane	Zerbaxa	+	—	+	—	None	Approved (2014/2019)	cUTI, cIAI (+ metronidazole), HAP/VAP	Inactive versus MBLs, *bla*_KPC_, *bla*_OXA-48_; resistance via AmpC mutations
**Avibactam**	Ceftazidime	Avycaz/Zavicefta	+	—	+	±	None	Approved (2015)	cIAI, cUTI, HAP/VAP (including *bla*_KPC_, *bla*_OXA-48_ producers)	No MBL activity; resistance via porin loss, efflux, or *blaKPC Ω*‐loop mutations.
**Avibactam**	Aztreonam	Emblaveo	+	—	+	±	None	Approved (2025)	cIAI	Ineffective against MBL + avibactam‐resistant serine *β*‐lactamase coproducers.
**Vaborbactam**	Meropenem	Vabomere/Vaborem	+	±	+	—	None	Approved (2017)	cUTI, cIAI, HAP/VAP (for KPC‐CRE)	Inactive versus MBLs and most *bla*_OXA-48_.
**Relebactam**	Imipenem–cilastatin	Recarbrio	+	—	+	±	None	Approved (2019)	cUTI, cIAI, HAP/VAP (for *blaKPC* producers and AmpC‐derepressed *P. aeruginosa*)	Weak versus *bla*_OXA-48_; no MBL activity.
**Durlobactam**	Sulbactam	Xacduro	+	—	+	+	PBP2 inhibition (synergistic with sulbactam′s PBP1/3 inhibition)	Approved (2023)	HAP/VAP caused by carbapenem‐resistant *A. baumannii* (CRAB)	Pathogen‐specific (CRAB). No MBL activity.
**Enmetazobactam**	Cefepime	Exblifep	+	—	±	±	None	Approved (2024)	cUTI (US), cUTI, and HAP/VAP (EU)	No MBL activity.
**Cefiderocol**	(Standalone)	Fetcroja/Fetroja	+	+	+	+	PBP3 inhibition (as a *β*‐lactam antibiotic)	Approved (2019)	cUTI, HAP/VAP (when limited/no alternative options)	Not a BLI. Resistance via siderophore receptor mutations or efflux.

#### 3.1.2. Clinical Impact and Spectrum Limitations

The fixed‐dose combination of amoxicillin and clavulanic acid (co‐amoxiclav) marked a paradigm shift in antimicrobial therapy. It successfully restored the clinical utility of aminopenicillins against a vast array of community‐acquired pathogens producing plasmid‐encoded Class A *β*‐lactamases, becoming a cornerstone for treating respiratory tract, urinary tract, skin and soft tissue, and intra‐abdominal infections [15].

However, the spectrum of clavulanic acid is inherently narrow. It is potent against many Class A enzymes but demonstrates weak activity against Class C (AmpC) and Class D (*bla*_OXA_‐type) *β*‐lactamases and possesses no activity against MBLs. Furthermore, its clinical use is constrained by a higher incidence of gastrointestinal adverse effects (notably diarrhea) compared with amoxicillin alone, which limits the maximum tolerable daily dose. Perhaps most importantly, its success paved the way for bacterial counter‐evolution, selecting for resistant mutants such as inhibitor‐resistant *bla*_TEM_ variants and organisms with derepressed AmpC production [15].

#### 3.1.3. Foundational Role and Legacy

Despite its limitations, the clinical and commercial success of co‐amoxiclav provided the indispensable proof‐of‐concept for the entire BLI strategy. It unequivocally demonstrated that pharmacologically inhibiting bacterial defense enzymes could resurrect the efficacy of existing antibiotic classes. This validation directly fueled the rational drug design efforts that led to the subsequent generations of inhibitors reviewed herein, from the synthetic penicillanic acid sulfones (TAZ) to the modern DBOs and boronates. Therefore, clavulanic acid is not merely a historical footnote but the foundational molecule whose mechanism and therapeutic rationale underpin the entire contemporary landscape of *β*‐lactamase inhibitor development [15].

### 3.2. TAZ: A First‐Generation Inhibitor With Evolving Applications

#### 3.2.1. Overview and Mechanism

TAZ is a penicillanic acid sulfone BLI whose structure features a 1,2,3‐triazol‐1‐yl group replacing an exocyclic methyl hydrogen (Figure 2) [17, 18]. It acts primarily by irreversibly inhibiting SBLs, protecting coadministered *β*‐lactams from hydrolysis. However, its utility is not absolute, as some Class A enzymes (e.g., *bla*_SHV-1_) can hydrolyze TAZ to a limited extent, highlighting an early vulnerability in this class of inhibitors [17, 18].

#### 3.2.2. Clinical Combinations and Spectrum

As summarized in Table 1, TAZ′s primary role has been in restoring the activity of piperacillin (PIP), an extended‐spectrum penicillin. The PIP/TAZ combination demonstrates a spectrum covering many *Enterobacterales* and *Pseudomonas aeruginosa*, but its inactivity against MBLs and variable activity against AmpC‐ and OXA‐type enzymes represents significant limitations [19–21].

A significant evolution in TAZ′s use is its partnership with the novel cephalosporin, ceftolozane (CEF). The CEF/TAZ combination was developed to enhance activity against MDR *P. aeruginosa* and extended‐spectrum *β*‐lactamase (ESBL)‐producing *Enterobacterales* [22–24]. As Table 1 indicates, while this combination improves upon PIP/TAZ against many Class A enzymes, it remains vulnerable to resistance mechanisms not covered by its spectrum.

#### 3.2.3. Resistance and Critical Analysis

The clinical value of CEF/TAZ is countered by emerging resistance, particularly in extensively drug‐resistant (XDR) *P. aeruginosa*. Mutations in chromosomal AmpC *β*‐lactamases, such as the G183V substitution, are key drivers of resistance, reducing porin permeability and enhancing affinity for specific PBPs [23, 25]. Furthermore, TAZ′s weak inhibition of AmpC enzymes and its lack of activity against carbapenemases (*bla*_KPC_ and *bla*_OXA-48_) and MBLs fundamentally limit the utility of both PIP/TAZ and CEF/TAZ against the most formidable Gram‐negative pathogens [19, 26–28]. This underscores a critical weakness of early‐generation BLIs: Their spectrum is easily circumvented by bacterial enzyme diversity and evolution.

#### 3.2.4. Clinical Applications and Comparative Outlook

Despite these limitations, TAZ‐based combinations remain clinically valuable within their niche. PIP/TAZ is a workhorse for community‐acquired intra‐abdominal infections and other polymicrobial infections, though it requires dosage adjustment in renal impairment and carries a noted risk of acute kidney injury, especially with concurrent vancomycin [20, 29, 30]. A recent randomized trial even suggested worse outcomes in septic patients receiving PIP/TAZ compared with cefepime (FEP), indicating its role may need re‐evaluation in specific severe infections [31].

In contrast, CEF/TAZ is a critical agent for MDR *P. aeruginosa* infections, having received U.S. Food and Drug Administration (FDA) approval for complicated urinary tract infections (cUTIs), complicated intra‐abdominal infections (cIAIs) (with metronidazole), and hospital‐acquired/ventilator–associated pneumonia (HAP/VAP) [31–35]. Its safety profile is generally favorable, with nausea and diarrhea being the most common adverse effects, though the risk of *Clostridioides difficile* infection persists [32, 36].

In summary, TAZ exemplifies the trajectory of BLI development: a first‐generation inhibitor that remains relevant through strategic new partnerships. However, its defined and relatively narrow spectrum of inhibition, coupled with well‐characterized resistance pathways, firmly establishes the need for the broader spectrum inhibitors that have followed.

### 3.3. Enmetazobactam (ENM): An Enhanced Penicillin Sulfone With Strategic Advantages

#### 3.3.1. Molecular Optimization Over TAZ

ENM is a novel penicillin sulfone BLI and structural analog of TAZ, distinguished by a strategically positioned methyl group that confers a neutral net charge (Figure 2) [13, 33, 34]. This zwitterionic nature, shared with its partner antibiotic FEP, enhances penetration through the Gram‐negative outer membrane, a critical pharmacokinetic improvement over earlier inhibitors [34].

Biophysically, ENM demonstrates broad‐spectrum activity against SBLs while maintaining scaffold integrity, contrasting with the fragmentation observed with SUL and TAZ [13]. Its enhanced cell‐based activity is likely mediated by improved permeability and reduced efflux pump susceptibility, underscoring a deliberate design to overcome intrinsic bacterial defenses [13].

#### 3.3.2. Cefepime/ENM: A Potent Carbapenem‐Sparing Regimen

The fixed‐dose combination of FEP/ENM (EXBLIFEP) received FDA approval in 2024, establishing a new carbapenem‐sparing option for MDR Gram‐negative infections [35, 37]. As detailed in Table 1, its spectrum primarily targets Classes A, C, and D *β*‐lactamases, showing superior in vitro activity against ESBL‐producing and *bla*_KPC_‐producing isolates compared with PIP/TAZ [13, 34, 37]. ENM can restore susceptibility to FEP from a baseline of 2%–98% for certain ESBL producers, highlighting its dramatic efficacy enhancement [19].

A key limitation, consistent with its profile, is the lack of activity against MBLs, rendering it ineffective against MBL‐producing pathogens [38]. Coadministration with aztreonam (ATM) has been proposed as a potential strategy to address this gap, though it requires further clinical validation [39].

#### 3.3.3. Clinical Positioning and Safety Profile

FEP/ENM is approved in the United States for cUTIs, including pyelonephritis, and in the EU for cUTIs, HAP, and VAP [35, 40]. Its role as a powerful, carbapenem‐sparing agent for suspected ESBL infections is a significant advancement in antimicrobial stewardship [34].

The combination is generally well tolerated. The most notable safety finding from clinical trials is the reversible elevation of liver enzymes, which was mostly low‐grade, with a single reported case of Grade 3 hepatotoxicity leading to treatment discontinuation [41]. Although animal studies suggest potential reproductive toxicity at high exposures, the teratogenic risk in humans remains undefined, and the cephalosporin component (FEP) is not generally considered teratogenic [40].

In summary, ENM represents a successful molecular optimization of the classic penicillin sulfone scaffold. Its partnership with cefepime creates a potent, targeted therapeutic that excels against a defined range of serine‐based resistance mechanisms. While its lack of MBL activity confines its utility, FEP/ENM stands as a valuable, carbapenem‐sparing tool specifically engineered to address the pervasive challenge of ESBLs and related enzymes in the clinical setting.

## 4. Diazabicyclooctane Based Inhibitors

DBOs mark a revolutionary shift in BLI design as the first non–*β*‐lactam, reversible covalent inhibitors. Their unique bicyclic structure enables potent, broad‐spectrum inhibition of SBLs through carbamylation of the active‐site serine. This class has rapidly expanded to include inhibitors with fine‐tuned spectra, dual PBP‐inhibitory activity, and oral prodrug formulations, addressing diverse clinical needs from hospital‐acquired infections to outpatient therapy [42].

### 4.1. AVI: Pioneering the Diazabicyclooctane Class With Strategic Spectrum Expansion

#### 4.1.1. A Paradigm Shift in BLI Design

AVI represents a revolutionary advance in BLI design as the first synthetic DBO compound approved for clinical use (Figure 2) [43, 44]. Its mechanism diverges from earlier inhibitors by forming a reversible, covalent bond with SBLs. As detailed in Table 1, AVI′s profile encompasses Classes A, C, and many Class D enzymes, effectively inhibiting ESBLs, AmpC cephalosporinases, and key carbapenemases like *bla*_KPC_ and *bla*_OXA-48_ [17, 45]. This marked a significant leap beyond the capabilities of TAZ‐based combinations.

A key strategic application is its combination with ATM. Since ATM is inherently stable against MBLs, the ATM/AVI combination leverages AVI to protect ATM from coproduced SBLs, creating a *β*‐lactam–based therapy with activity against MBL‐producing pathogens [46].

#### 4.1.2. Ceftazidime (CAZ)/AVI: A Cornerstone for KPC and OXA‐48

The combination of CAZ with AVI (CAZ/AVI) was the first *β*‐lactam therapy specifically designed for CRE [30]. Its strength lies in treating infections caused by *bla*_KPC_ and *bla*_OXA-48_ producers [43, 47, 48].

However, CAZ/AVI′s limitations are significant. It lacks activity against MBLs, and efficacy can be compromised by porin mutations (OprD loss), efflux pump overexpression, and AmpC overexpression in *P. aeruginosa* [49, 50]. Crucially, resistance can arise from mutations within the *β*‐lactamase *Ω*‐loop itself [48, 51]. Furthermore, CAZ/AVI provides unreliable coverage against *Acinetobacter baumannii* and anaerobic bacteria [43].

Clinically, CAZ/AVI is approved for cIAIs, cUTIs, and HAP/VAP, demonstrating efficacy in bacteremia and pneumonia [43, 52, 53]. Its safety profile is generally favorable, though a positive direct Coombs test is a notable side effect [54].

#### 4.1.3. ATM/AVI: Strategically Countering the MBL Threat

The approval of ATM/AVI represents a strategic solution to MBL‐mediated resistance. This combination exploits a unique synergistic mechanism: ATM, a monobactam, is inherently stable to hydrolysis by MBLs. However, MBL‐producing pathogens often coproduce SBLs that can hydrolyze ATM. AVI potently inhibits these coproduced SBLs, thereby protecting ATM and allowing it to reach its PBP target. As Table 1 confirms, this synergy makes ATM/AVI a critical option for pathogens producing MBLs (e.g., *bla*_NDM_) alongside other *β*‐lactamases [55, 56].

This makes ATM/AVI a last‐resort option for daunting MDR Gram‐negative infections. Nonetheless, resistance is emerging, primarily through mutations in PBP3 [47, 50]. Approved for cIAIs, ATM/AVI′s role is narrow and should be reserved to preserve its efficacy [57].

In summary, AVI reshaped the BLI landscape through broader spectrum, reversible inhibition. Its combinations, CAZ/AVI and ATM/AVI, provide a powerful strategy: the former addresses serine carbapenemases, whereas the latter counters the MBL challenge. Despite this, both face evolving resistance, underscoring the need for continuous innovation.

### 4.2. Relebactam (REL): A Diazabicyclooctane With a Focus on Pseudomonas

#### 4.2.1. Structural Nuance and Spectrum

REL is a diazabicyclooctane BLI structurally akin to AVI, distinguished by a piperidine ring substitution that fine‐tunes its properties (Figure 2) [58]. Its inhibitory spectrum is robust against Classes A carbapenemases (*bla*_KPC_) and C (AmpC) enzymes but demonstrates weaker activity against Class D (e.g., *bla*_OXA-48_) and no activity against MBLs (Table 1) [38, 59, 60]. This profile positioned it as a candidate to resurrect the activity of imipenem against specific resistant pathogens.

#### 4.2.2. Imipenem/REL: Resurrecting a Classic Carbapenem

The combination of imipenem–cilastatin with REL (IMI/REL) was developed to counter carbapenem‐resistant *P. aeruginosa* and *bla*_KPC_‐producing *Enterobacterales* [60, 61]. REL potently inhibits AmpC and *bla*_KPC_ enzymes, effectively protecting imipenem from hydrolysis and restoring its activity against many nonsusceptible strains [61–63]. A key differentiator is its reliable activity against *P. aeruginosa*, including strains with derepressed AmpC, a setting where some other BLI combinations may be less reliable [49, 64].

However, direct comparative studies have shown that IMI/REL can exhibit higher MICs against some carbapenemase‐producing *Enterobacteriaceae* compared with meropenem/vaborbactam (MER/VAB) and CAZ/AVI, suggesting that VAB and AVI may have more potent inhibitory strength in certain contexts [43]. Its well‐defined limitations, incomplete inhibition of *bla*_OXA-48_, and inactivity against MBLs restrict its utility in geographies where these enzymes are prevalent [59, 60].

#### 4.2.3. Clinical Role and Safety

IMI/REL is approved for cUTIs, cIAIs, and HAP/VAP [60, 65, 66]. Its clinical significance is underscored by a randomized trial where it demonstrated efficacy comparable with colistin for carbapenem‐nonsusceptible infections but with a vastly superior safety profile, notably avoiding the nephrotoxicity associated with polymyxins [67]. The most common adverse effects are gastrointestinal (nausea, diarrhea) and headache, which are generally manageable [68].

Despite its effectiveness, resistance during therapy, particularly in *bla*_KPC_‐producing *K. pneumoniae*, has been documented, often involving porin mutations in conjunction with KPC overexpression [69].

In summary, REL successfully leverages the DBO scaffold to create a valuable niche. The IMI/REL combination is a critically important option for serious infections caused by difficult‐to‐treat *P. aeruginosa* and *bla*_KPC_‐producing *Enterobacterales*, offering a much‐needed, less toxic alternative to colistin. Although it may not be the most potent inhibitor against all serine enzymes in vitro, its proven clinical efficacy and safety in its intended niche secure its role in the antimicrobial armamentarium.

### 4.3. DUR: A Targeted Strategic Weapon Against *Acinetobacter*

#### 4.3.1. Structural and Mechanistic Innovation

DUR is a next‐generation diazabicyclooctane BLI whose structure is distinguished by an endocyclic double bond and a C‐3 methyl group (Figure 2), enhancing its reactivity and binding affinity compared with earlier compounds in its class [70–72]. Its mechanism involves reversible covalent carbamylation of SBLs, similar to AVI, but it is specifically optimized for greater potency against Class D OXA‐type carbapenemases, a hallmark resistance mechanism in *A. baumannii* [73, 74].

#### 4.3.2. SUL/DUR: A Synergistic Dual‐Action Therapy

The combination of SUL with DUR (SUL/DUR) represents a paradigm shift in the treatment of carbapenem‐resistant *A. baumannii* (CRAB). This copackaged therapy leverages a unique dual mechanism: SUL acts as a foundational antibiotic by inhibiting PBP1a/1b and PBP3, whereas DUR provides a dual benefit by potently inhibiting the broad spectrum of SBLs that degrade SUL and independently inhibiting PBP2 [70, 75, 76]. This comprehensive targeting of essential PBPs and *β*‐lactamase defense creates a highly synergistic effect, maximizing antibacterial activity against CRAB [73].

As Table 1 illustrates, SUL/DUR′s spectrum is precisely tailored for *Acinetobacter*, effectively neutralizing Classes A, C, and D enzymes prevalent in this pathogen [77]. Its excellent intrapulmonary penetration further validates its approved indication for HAP and VAP caused by susceptible CRAB strains [75, 78].

#### 4.3.3. Clinical Niche and Emerging Resistance

SUL/DUR filled a critical therapeutic void, demonstrating noninferiority to colistin for CRAB infections while offering a significantly superior safety profile, with headache and nausea being the most common adverse events rather than nephrotoxicity [79]. Its ability to inhibit over 95% of CRAB isolates, including colistin‐resistant strains, positions it as a first‐line targeted therapy [80].

However, its Achilles heel is a complete lack of activity against MBLs, rendering it ineffective against the minority of CRAB strains that produce these enzymes [80]. Resistance, while currently rare, primarily emerges via mutations in the *ftsI* gene (encoding PBP3) or the acquisition of MBLs, highlighting the need for ongoing susceptibility testing and stewardship to preserve its efficacy [77, 80].

In summary, DUR is not a broad‐spectrum inhibitor but a precision tool. Its development and combination with SUL exemplify a successful pathogen‐focused strategy. SUL/DUR provides a much‐needed, effective, and safer alternative to polymyxins for CRAB, addressing a paramount challenge in hospital‐acquired infections. Its defined spectrum underscores that the future of BLI development may lie not only in breadth but also in strategic depth against high‐priority targets.

### 4.4. NAC: A Dual‐Action Diazabicyclooctane With Strategic Partnerships

#### 4.4.1. A Dual‐Mechanism Innovation

NAC is a diazabicyclooctane BLI whose structure is optimized with a carbamoyl side chain, conferring enhanced potency against SBLs compared with AVI (Figure 2) [81]. Its defining characteristic is a dual mechanism of action: it functions as a potent BLI against Classes A (*bla*_KPC_ and ESBLs), C (AmpC), and some Class D enzymes, while also independently binding to PBP2 in Enterobacterales [82, 83]. This intrinsic antibacterial activity, rare among BLIs, creates a powerful synergistic effect when combined with a partner *β*‐lactam, potentially leading to enhanced bacterial killing and a higher barrier to resistance [82, 84].

#### 4.4.2. Strategic Clinical Combinations

NAC′s versatility is demonstrated by its parallel development in three distinct intravenous combinations, each designed to address a specific resistance profile, as summarized in Table 2.

**Table 2:** *β*‐Lactam/*β*‐lactamase inhibitor combinations in clinical development.

Inhibitor	Partner *β*‐lactam	Inhibition spectrum (*β*‐lactamase class)				Intrinsic PBP inhibition (by BLI alone)	Clinical status and year	Key investigational indications	Key limitations
		A	B	C	D				
**Taniborbactam**	Cefepime	+	+	+	+	None	Phase 3	cUTI (including pyelonephritis), HAP/VAP	Weak against *bla*_IMP_‐type MBLs.
**Zidebactam**	Cefepime	+	±	+	+	*β*‐lactam enhancer via PBP2 binding	Phase 3	MDR/XDR *P. aeruginosa*, CRAB, CRE	Complex, nonstandard MOA; variable activity versus *S. maltophilia*, *Proteus*, *Serratia*.
**Nacubactam**	Cefepime	+	—	+	±	PBP2 inhibition	Phase 3	cUTI, cIAI, HAP/VAP	No activity against MBL‐producing bacteria
**Nacubactam**	Meropenem	+	—	+	±	PBP2 inhibition	Phase 3	cUTI, HAP/VAP	No activity against MBL‐producing bacteria
**Nacubactam**	Aztreonam	+	+	+	+	PBP2 inhibition	Phase 3	Serious bacterial infections (targeting MBL producers)	Relies on aztreonam′s MBL stability; potential resistance via PBP mutations.
**Xeruborbactam**	Tebipenem	+	+	+	+	Not fully defined (ultrabroad spectrum)	Phase 1	cUTI (oral step‐down), *M. abscessus* pulmonary disease (investigational)	Early development stage.
**ETX0282**	Cefpodoxime proxetil	+	—	+	+	Yes: PBP2 inhibition (from ETX1317)	Phase 1	cUTI (oral outpatient therapy)	Very low oral bioavailability in humans (major PK challenge).
**ARX-1796**	Ceftibuten	+	—	+	+	None	Phase 1	cUTI (oral outpatient therapy)	Oral‐only; inherits avibactam′s lack of MBL activity.
**Ledaborbactam etzadroxil (VNRX-7145)**	Ceftibuten	+	—	+	+	None	Phase 1	CUTI (oral outpatient therapy)	Oral‐only; PK challenges of oral boronate BLIs.

##### 4.4.2.1. Cefepime/Nacubactam (FEP/NAC)

This combination leverages the synergistic PBP targeting of NAC (PBP2) and FEP (PBP3) [83]. It exhibits robust in vitro activity against MDR *Enterobacterales*, including CRE‐ and ESBL‐producers [85, 86]. Intriguingly, FEP/NAC has demonstrated a notable inhibition rate (80.3%–93.3%) against some MBL‐producing strains, an effect likely attributable to its dual PBP inhibition bypassing enzymatic resistance rather than inhibiting the MBL itself [87]. This combination is advancing in Phase 3 trials for cUTIs, HAP/VAP, and cIAIs [88].

##### 4.4.2.2. Meropenem/Nacubactam (MER/NAC)

The MER/NAC combination aims to fortify meropenem against CRE [84, 89]. It is active against serine carbapenemases like *bla*_KPC_ and *bla*_OXA-48_ but shares the common limitation of diminished efficacy against MBLs [64, 81]. Its exploration in preclinical models of *Mycobacterium abscessus* complex infections suggests a potential application beyond traditional Gram‐negative pathogens [82]. Also in Phase 3 development, its safety profile indicates phlebitis as a common adverse event [19, 88].

##### 4.4.2.3. Aztreonam/Nacubactam (ATM/NAC)

The ATM/NAC pairing represents one of the most comprehensive strategic approaches to MBL‐producing pathogens [90]. ATM is stable against MBL hydrolysis, whereas NAC protects it from coproduced SBLs. The addition of NAC′s anti‐PBP2 activity provides a third mechanistic layer. This makes ATM/NAC highly active against pathogens producing MBLs (e.g., *bla*_NDM_ and *bla*_IMP_) in conjunction with SBLs and *bla*_OXA-48_ [90, 91]. This combination is also undergoing Phase 3 evaluation for serious bacterial infections [92, 93].

In summary, NAC′s principal innovation is its dual‐action capability, which provides a mechanistic advantage beyond pure enzyme inhibition. Its development across three distinct combinations, FEP/NAC, MER/NAC, and ATM/NAC, exemplifies a strategic, portfolio‐based approach to MDR Gram‐negative infections. While FEP/NAC and MER/NAC target serine‐based resistance with an added PBP2 punch, ATM/NAC is poised to become a cornerstone therapy for the most daunting MBL‐producing pathogens. This multifaceted strategy solidifies NAC′s role as a pivotal and versatile candidate in the next generation of BLI‐based therapies.

### 4.5. Oral DBO Prodrugs

#### 4.5.1. ARX‐1796: An Oral Prodrug for AVI

##### 4.5.1.1. An Oral Prodrug for AVI

ARX‐1796 represents a significant strategic advancement as an orally bioavailable prodrug of AVI (Figure 2) [94]. Its development addresses a critical therapeutic gap: the lack of effective oral regimens for MDR Gram‐negative infections. By enabling oral administration, ARX‐1796 has the potential to transform patient care, facilitating early discharge from hospitals and managing complex infections in an outpatient setting.

##### 4.5.1.2. Ceftibuten (CFB)/ARX‐1796: A Promising Oral Combination

The primary clinical candidate is the combination of ARX‐1796 with the oral cephalosporin CFB. Preclinical data for CFB/ARX‐1796 is highly promising, demonstrating potent activity against a broad range of *β*‐lactamase‐producing Enterobacterales, including ESBL, AmpC, *bla*_KPC_, and *bla*_OXA-48_‐like producers (Table 2) [94, 95]. Notably, this combination has shown lower minimum inhibitory concentrations (MICs) compared with other oral antibiotic options, suggesting superior potential efficacy [95].

This combination is currently undergoing Phase 1 clinical trials, with an initial focus on treating cUTIs, including acute pyelonephritis [95, 96]. Its success could establish the first oral therapy specifically designed for highly resistant pathogens in the community.

##### 4.5.1.3. Expanding the Horizon: Potential Beyond *Enterobacterales*

Intriguingly, the utility of ARX‐1796 may extend beyond traditional Gram‐negative pathogens. Research has explored its combination with the oral carbapenem tebipenem pivoxil (TEB) against *M. abscessus*, a notoriously drug‐resistant organism. In a mouse model of lung infection, the TEB/ARX‐1796 combination achieved over 90% reduction in bacterial burden, highlighting a novel application for this BLI platform against nonenteric bacteria [96–98].

In summary, ARX‐1796 is more than a mere prodrug; it is a strategic enabler of oral therapy for resistant infections. While its primary path is with CFB against MDR Enterobacterales, its potential application with TEB against *M. abscessus* illustrates a broader therapeutic versatility. The success of this oral platform could mark a paradigm shift from inpatient to outpatient management of serious resistant infections, significantly impacting antibiotic stewardship and healthcare systems.

#### 4.5.2. ETX0282: The Quest for an Advanced Oral BLI

##### 4.5.2.1. A Diazabicyclooctane Prodrug for Oral Delivery

ETX0282 represents a significant endeavor to develop an orally bioavailable DBO‐based BLI. It is a prodrug of the active compound ETX1317, a regioisomer of DUR, engineered with an (R)‐2‐fluoroacetate group to enhance oral absorption (Figure 2) [99, 100]. Its mechanism of action aligns with other DBOs, involving reversible covalent inhibition of SBLs via carbamylation [101]. A notable feature is its intrinsic activity through binding to PBP2, contributing to a synergistic effect when combined with a partner *β*‐lactam antibiotic [102].

##### 4.5.2.2. Cefpodoxime/ETX0282: A Potential Outpatient Game‐Changer

The combination of ETX0282 with the oral cephalosporin cefpodoxime proxetil (CPD) is pioneering as the first oral *β*‐lactam/BLI combination to enter Phase 1 clinical trials [37, 103]. Preclinical data are compelling, demonstrating potent in vitro activity against a broad range of MDR *Enterobacterales*, including strains producing ESBLs, *bla*_KPC_, and *bla*_OXA-48_‐like carbapenemases [3, 95, 104, 105]. As summarized in Table 2, its spectrum covers Classes A, C, and D enzymes, but it shares the common limitation of lacking activity against MBLs [100].

The primary clinical vision for CPD/ETX0282 is to serve as an oral step‐down or outpatient therapy for cUTIs caused by resistant pathogens, potentially preventing hospitalizations and reducing the reliance on intravenous antibiotics [95, 99].

##### 4.5.2.3. Developmental Challenges and Outlook

Despite its promising preclinical profile, the development of ETX0282 has faced a critical hurdle. Phase 1 trials revealed unexpectedly low oral bioavailability in humans, a stark contrast to the approximately 78% bioavailability observed in preclinical animal models [100]. This pharmacokinetic challenge has likely impeded its clinical progression.

The combination was reported to be well tolerated, with only mild to moderate gastrointestinal adverse events like vomiting noted, and no serious safety signals emerged [95, 99]. However, the bioavailability issue remains the central obstacle to its future.

In summary, ETX0282/CPD embodies the highly sought‐after goal of an effective oral regimen for MDR Gram‐negative infections. Its advanced preclinical spectrum against key serine carbapenemases positioned it as a potential breakthrough for outpatient management. However, its developmental trajectory underscores a recurrent challenge in drug discovery: the difficult translation of promising pharmacokinetic data from animals to humans. Its fate highlights that for oral BLIs, achieving sufficient systemic exposure is as critical as designing potent enzyme inhibition.

## 5. Boronate‐Based Inhibitors

Boronate‐based inhibitors employ a distinct chemical strategy, utilizing a boron atom to mimic the tetrahedral transition state of *β*‐lactam hydrolysis. This mechanism grants them exceptional affinity for SBLs, particularly *bla*_KPC_ carbapenemases. Advances in this scaffold have yielded agents with expanded spectra, including the pioneering broad‐spectrum inhibitor TAN, which overcomes the historical challenge of MBL inhibition and oral prodrugs designed for outpatient use [67].

### 5.1. VAB: A Boronic Acid Inhibitor With Precision KPC Activity

#### 5.1.1. A Unique Non–*β*‐Lactam Scaffold

VAB is a cyclic boronic acid–derived BLI that stands apart structurally and mechanistically from both penicillanic sulfones and DBOs (Figure 2) [38, 106]. Its defining feature is the boron atom, which forms a reversible covalent bond with the catalytic serine residue of SBLs, mimicking the tetrahedral transition state of *β*‐lactam hydrolysis. This unique mechanism results in exceptionally potent inhibition of *bla*_KPC_ enzymes, surpassing earlier BLIs in this specific role [38, 107, 108].

#### 5.1.2. MER/VAB: A Premier Choice for KPC‐CRE

The combination of meropenem with VAB (MER/VAB) was specifically engineered to address the rising threat of KPC‐producing CRE. As detailed in Table 1, its spectrum is highly focused, showing outstanding activity against Class A enzymes, including *bla*_KPC_, but offering no reliable coverage against Class B or most Class D (e.g., *bla*_OXA-48_‐like) carbapenemases [106, 109].

This targeted profile makes MER/VAB a first‐line therapeutic option for infections caused by *bla*_KPC_‐producers, demonstrating superior efficacy in some real‐world studies compared with other advanced regimens like CAZ/AVI and IMI/REL [110, 111]. Its utility extends to rare serine carbapenemases like *bla*_SME_ in *Serratia marcescens* [112]. However, its effectiveness is vulnerable to the emergence of resistance mechanisms commonly seen in *bla*_KPC_‐producing strains, including porin mutations coupled with *β*‐lactamase overexpression or enhanced efflux pump activity [113–115].

#### 5.1.3. Clinical Efficacy and Safety Positioning

MER/VAB is approved for cUTIs, cIAIs, and HAP/VAP caused by susceptible *Enterobacteriaceae* [106, 116]. Clinical data confirm its superior outcomes over PIP/TAZ in cUTIs, with a significant advantage in its safety profile, particularly a lower incidence of nephrotoxicity compared with older, last‐resort therapies [117]. The most common adverse events are gastrointestinal and are generally well tolerated [118].

In summary, VAB exemplifies a precision medicine approach in the BLI arsenal. It does not boast the broadest spectrum, but its unparalleled potency against *bla*_KPC_ enzymes makes the MER/VAB combination a cornerstone for managing *bla*_KPC_–CRE infections. Its favorable renal safety profile further solidifies its role as a key agent in the treatment of these challenging infections, highlighting that supreme efficacy against a specific, high‐priority target can be more valuable than moderate activity against a wider range.

### 5.2. TAN: A Broad‐Spectrum Pioneer Against MBLs

#### 5.2.1. A Landshift in BLI Spectrum

TAN represents a quantum leap in BLI development as one of the first broad‐spectrum, bicyclic boronate inhibitors capable of targeting both SBLs and MBLs (Figure 2) [92, 119, 120]. Its mechanism involves mimicking the tetrahedral intermediate of *β*‐lactam hydrolysis, effectively inhibiting a wide range of enzymes, including *bla*_KPC_ (Class A), AmpC (Class C), *bla*_OXA-48_ (Class D), and critically, MBLs like *bla*_VIM_ and many *bla*_NDM_ variants [119, 121]. This profile, summarized in Table 2, positions TAN as a potential universal protector for partner *β*‐lactams, directly addressing the longstanding MBL challenge.

#### 5.2.2. Cefepime/TAN: A Potential Pan‐Resistance Agent

The combination of cefepime with TAN (FEP/TAN) is a highly promising therapeutic candidate currently in advanced Phase 3 trials [88, 122]. Preclinical and early clinical data suggest it possesses one of the broadest spectra among novel BLI combinations. It demonstrates potent activity against a daunting range of MDR pathogens, including CRE producing *bla*_KPC_, *bla*_NDM_, *bla*_VIM_, and *bla*_OXA-48_, as well as carbapenem‐resistant *P. aeruginosa* and *Stenotrophomonas maltophilia* [119, 123–125].

Comparative studies have shown FEP/TAN to be more active than other advanced regimens like CAZ/AVI and MER/VAB against MBL‐ and *bla*_OXA-48_‐producing strains, filling a critical therapeutic void [126]. However, its Achilles′ heel is a lack of activity against IMP‐type MBLs, and resistance can emerge through complex mechanisms including IMP production, PBP3 mutations, and efflux pump overexpression [37, 119].

#### 5.2.3. Clinical Prospects and Developmental Status

FEP/TAN is being developed for the treatment of cUTIs, including pyelonephritis, and HAP/VAP [88]. Recent trial data indicate it may be more effective than meropenem in treating cUTIs while maintaining a similar safety profile, with headache and gastrointestinal disturbances being the most common side effects [127]. Its pharmacokinetics support a 2‐h infusion regimen, with dosage adjustments required in renal impairment [125, 128].

In summary, TAN is a trailblazing molecule that shatters the historical boundary between SBL and MBL inhibition. The FEP/TAN combination holds the potential to become a first‐line, empiric therapy for the most feared MDR Gram‐negative infections, particularly in regions with a high prevalence of MBLs. Its development marks a pivotal moment in anti‐infective research, offering a glimpse into a future where a single *β*‐lactam/BLI combination could reliably treat the vast majority of highly resistant pathogens.

### 5.3. Ledaborbactam: Pursuing the Oral BLI Holy Grail

#### 5.3.1. A Targeted Oral Inhibitor in Development

Ledaborbactam etzadroxil (LED; VNRX‐7145) is an investigational, orally bioavailable BLI specifically designed to combat resistant Gram‐negative infections in the outpatient setting (Figure 2) [129]. Its preclinical profile indicates potent activity against key SBLs, including Class A carbapenemases (*bla*_KPC_), Class C (AmpC), and Class D (OXA‐type) enzymes, positioning it as a targeted oral solution for MDR Enterobacterales [104, 130].

#### 5.3.2. CFB/Ledaborbactam Etzadroxil: A Potential Outpatient Revolution

The primary clinical candidate is the combination of LED etzadroxil with the oral cephalosporin CFB. Preclinical data for CFB/LED is highly promising, demonstrating efficacy against a broad range of Enterobacterales producing ESBLs, *bla*_KPC_, *bla*_OXA-48_‐like, and AmpC *β*‐lactamases [130]. As outlined in Table 2, this spectrum would directly address the critical gap of effective oral therapies for infections historically requiring intravenous agents like carbapenems or newer *β*‐lactam/BLI combinations.

The potential impact of a successful CFB/LED regimen cannot be overstated. It could revolutionize management by enabling early discharge, facilitating prolonged outpatient therapy, and providing a carbapenem‐sparing option for MDR cUTIs and other suitable indications [130]. This would significantly alleviate healthcare burdens and improve patient quality of life.

#### 5.3.3. Developmental Status and Critical Challenges

Currently in Phase 1 clinical trials, the future of CFB/LED hinges on demonstrating adequate oral bioavailability, safety, and tolerability in humans [95]. The development pathway for oral BLIs is fraught with challenges, as evidenced by the setback with ETX0282, where promising animal pharmacokinetics failed to translate to humans. Furthermore, like other advanced BLIs in its class, its utility will be limited against MBL‐producing pathogens, an inherent constraint of its mechanism.

In summary, LED represents the continued, vigorous pursuit of an effective oral BLI. The CFB/LED combination embodies the highly sought‐after goal of managing complex MDR infections outside the hospital. While its targeted spectrum against major serine carbapenemases is precisely what is needed, its ultimate success is entirely contingent upon overcoming the formidable pharmacokinetic and developmental hurdles that have historically plagued the field of oral BLI development.

## 6. Inhibitors With Novel or Dual Mechanisms

Moving beyond traditional enzyme inhibition, the latest generation of BLIs incorporates innovative mechanisms to overcome resistance. This includes agents that not only inhibit *β*‐lactamases but also directly bind PBPs, creating synergistic enhancer effects. Additionally, ultrabroad‐spectrum inhibitors capable of neutralizing all Ambler classes have emerged, representing a significant leap toward universal *β*‐lactam protectors.

### 6.1. ZID: A Dual‐Action *β*‐Lactam Enhancer With Broad Potential

#### 6.1.1. The *β*‐Lactam Enhancer Mechanism

ZID is a novel bicyclo‐acyl hydrazide BLI that operates through a unique dual‐action mechanism, distinguishing it from conventional inhibitors (Figure 2) [66, 131, 132]. Its activity is twofold: It functions as a *β*‐lactamase inhibitor against Classes A and C enzymes, and more uniquely, it acts as a *β*‐lactam enhancer by binding to PBP2. This PBP2 inhibition powerfully synergizes with PBP3‐targeting *β*‐lactams like FEP, creating a potent, targeted bactericidal combination that attacks the bacterial cell wall at two critical points (Table 2) [131].

#### 6.1.2. Cefepime/ZID: Targeting a Broad MDR Spectrum

The combination of cefepime with ZID (FEP/ZID) is a promising advanced therapeutic candidate currently in Phase 3 clinical trials [88]. Its enhanced PBP targeting translates into an impressively broad in vitro spectrum. FEP/ZID demonstrates potent activity against a wide range of MDR and XDR Gram‐negative pathogens, including *P. aeruginosa*, Enterobacterales producing *bla*_KPC_ and *bla*_OXA-48_‐like carbapenemases, and notably, *A. baumannii* [131, 133]. Its activity against CRAB is particularly significant, with studies suggesting it may be more potent than several other advanced BLI combinations in this challenging pathogen [131].

However, its spectrum is not universal. FEP/ZID shows limited activity against *S. maltophilia*, *Proteus*, and *Serratia*, indicating its utility is focused on key ESKAPE pathogens rather than all nonfermenters [88]. Furthermore, while it shows some activity against certain MBL producers, this is likely attributable to its enhanced PBP targeting bypassing resistance rather than direct enzyme inhibition, as it is not a potent MBL inhibitor [133].

#### 6.1.3. Clinical Promise and Future Role

Early clinical reports, including a case of successful treatment for XDR *P. aeruginosa* sepsis, underscore its potential as a last‐line option where other therapies fail [134]. The combination has been well tolerated in studies, with no significant drug‐related side effects reported during extended courses, suggesting a favorable safety profile [133].

In summary, ZID represents a paradigm shift from pure enzyme inhibition to a more holistic enhancer strategy. The FEP/ZID combination leverages synergistic PBP targeting to achieve a broad spectrum that encompasses the most pressing MDR pathogens, including CRAB and XDR *P. aeruginosa*. While its development is ongoing, its unique mechanism and promising preclinical and early clinical data position it as a potential cornerstone for the treatment of polyclonal, carbapenem‐resistant infections, filling a critical gap that many narrower‐spectrum BLIs cannot address.

### 6.2. XER: An Ultrabroad‐Spectrum Inhibitor With Versatile Potential

#### 6.2.1. Unprecedented Breadth of Activity and Mechanism of Action

XER emerges as a potentially best‐in‐class ultrabroad‐spectrum BLI, demonstrating potent in vitro activity against all four Ambler classes (A, B, C, and D) of *β*‐lactamases [135]. This comprehensive coverage, detailed in Table 2, positions it as one of the most promising inhibitors in development, capable of protecting partner antibiotics from virtually all clinically relevant enzymatic resistance mechanisms in Gram‐negative bacteria. Its remarkable spectrum is attributed to a novel mechanism centered around its bridged bicyclic structure (Figure 2). While the precise molecular details are still being elucidated, preliminary data suggest XER acts as a potent, reversible covalent inhibitor. For SBLs, it is proposed to form a stable acyl–enzyme complex. For MBLs, its mechanism likely involves effective chelation or displacement of the zinc ions in the active site, mimicking the hydrolysis transition state. Its activity extends beyond *Enterobacterales* to include notoriously difficult‐to‐treat pathogens like *A. baumannii* and *P. aeruginosa*, a combination rarely achieved [136].

#### 6.2.2. A Platform for Innovative Formulations and Combinations

A key advantage of XER is its favorable pharmacokinetic profile, characterized by low plasma clearance and a long half‐life, making it suitable for both intravenous and oral administration [115]. This versatility is being exploited through the development of QPX7831, an oral prodrug of XER, which could enable powerful outpatient parenteral antibiotic therapy (OPAT) for resistant infections [8, 137].

The potential applications of XER are remarkably diverse. It is being investigated in an all‐oral regimen with tebipenem pivoxil (TEB) for *M. abscessus* pulmonary disease, a groundbreaking approach for this resistant mycobacterium [138]. Furthermore, it shows synergistic potential even with last‐resort antibiotics like cefiderocol, enhancing their activity against highly resistant *A. baumannii* strains, which suggests a role in salvage combination therapies [139]. Its broad utility is underscored by its demonstrated ability to enhance the activity of already‐approved *β*‐lactam/BLI combinations, potentially resurrecting their efficacy against strains that have developed resistance [140].

#### 6.2.3. Developmental Status and Future Outlook

Currently in Phase 1 clinical trials, XER′s early data on tolerability and pharmacokinetics are promising [8, 137]. Its development pathway is uniquely flexible, targeting not only traditional Gram‐negative infections but also venturing into the realm of nontuberculous mycobacteria.

In summary, XER is not merely another BLI; it is a versatile therapeutic platform. Its novel mechanism, enabling ultrabroad‐spectrum inhibition across all *β*‐lactamase classes, addresses the core challenge of enzymatic diversity. Coupled with its favorable pharmacokinetic properties and synergistic potential, XER opens doors to novel treatment modalities, from oral step‐down therapy to potent combination regimens for the most recalcitrant infections. If its early promise holds in advanced trials, XER could fundamentally expand the boundaries of how we approach antimicrobial therapy.

## 7. Cefiderocol: A Siderophore Cephalosporin Bypassing Resistance

### 7.1. A Trojan Horse Mechanism Against Gram‐Negative Pathogens

Cefiderocol is a pioneering siderophore cephalosporin that represents a novel class of antibiotics, circumventing traditional resistance mechanisms through a unique Trojan horse strategy (Figure 2) [141–143]. Its structure is engineered to mimic natural siderophores, iron‐scavenging molecules that bacteria actively import. This allows cefiderocol to hijack active iron‐transport systems (TonB‐dependent transporters), facilitating its entry into the periplasmic space even in bacteria with porin deficiencies or upregulated efflux pumps, two of the most common resistance pathways in Gram‐negative pathogens [142, 143].

### 7.2. An Unparalleled Spectrum of Activity

This innovative mechanism of entry grants cefiderocol one of the broadest spectra of activity against modern MDR Gram‐negative bacteria. It demonstrates potent efficacy against all major nonfermenting rods, including *P. aeruginosa*, *A. baumannii*, and *S. maltophilia*, as well as *Enterobacterales* [143–145]. Crucially, and as detailed in Table 1, cefiderocol remains stable against hydrolysis by a vast range of *β*‐lactamases, encompassing both serine carbapenemases (*bla*_KPC_ and *bla*_OXA-48_‐like) and metallo‐*β*‐lactamases (*bla*_NDM_, *bla*_VIM_, and *bla*_IMP_), making it a true last‐resort option for carbapenem‐resistant infections [144]. Its main spectrum limitation is a lack of activity against Gram‐positive and anaerobic bacteria [146].

### 7.3. Clinical Applications and Emerging Resistance

Cefiderocol received FDA approval for cUTIs and is a critical option for infections where limited or no alternatives exist [147]. Its utility extends to HAP/VAP, where it has been established as an effective and well‐tolerated treatment, even in the context of the CREDIBLE‐CR study, which noted a mortality signal requiring careful interpretation in specific patient populations [143, 145, 148].

However, the relentless pressure of bacterial evolution is evident. Resistance to cefiderocol, though still relatively rare, is emerging through diverse mechanisms. These include mutations in the siderophore receptors it exploits for entry (*piuA* and *pirA*), loss of porins, overexpression of specific efflux pumps (e.g., AdeABC and MexAB‐OprM), and surprisingly, the production of certain *β*‐lactamases like *bla*_PER-1_ and some *bla*_NDM_ variants that can slowly hydrolyze the drug [143].

In summary, cefiderocol is more than a new antibiotic; it is a conceptual breakthrough in overcoming bacterial permeability barriers. Its siderophore‐based transport system provides a robust workaround for common resistance mechanisms, granting it a uniquely broad spectrum against the most daunting MDR Gram‐negative pathogens, including those producing MBLs. While emerging resistance underscores the need for prudent use, cefiderocol remains an indispensable last‐line agent in the global fight against antimicrobial resistance, effectively serving as a safety net when all other *β*‐lactam options have failed.

## 8. Conclusion and Future Perspectives

The escalating crisis of antimicrobial resistance, driven predominantly by the relentless evolution and dissemination of *β*‐lactamases, demands an urgent and sophisticated response. This review has systematically charted the remarkable scientific journey from the first‐generation penicillin sulfone inhibitors to the contemporary era of structurally advanced, broad‐spectrum agents. This evolution reflects a critical paradigm shift from serendipitous discovery to rational, structure‐guided drug design, yielding innovative chemotypes such as DBOs (e.g., AVI and REL), boronic acid derivatives (e.g., VAB), and other novel scaffolds. The strategic pairing of these inhibitors with complementary *β*‐lactam antibiotics has successfully created targeted therapies against specific resistance threats, exemplified by CAZ/AVI for *bla*_KPC_‐producing *Enterobacterales* and the ingenious ATM‐based combinations (e.g., ATM/AVI) that circumvent MBL resistance through a synergistic two‐pronged mechanism.

Despite these advancements, the battle is intrinsically asymmetric. The development of each new inhibitor, including the promising ultrabroad‐spectrum agents like TAN and XER, is met with the inevitable emergence of bacterial countermeasures. Resistance increasingly arises from nonenzymatic pathways, porin mutations, efflux pump overexpression, and alterations in PBPs, highlighting that enzymatic inhibition alone is insufficient. This underscores the necessity for a multifaceted and forward‐looking strategy. The future of BLI development must be rooted in a deep understanding of bacterial evolution and resistance epidemiology. Key priorities include the application of artificial intelligence and computational biology to anticipate resistance and design next‐generation inhibitors; the pursuit of pathogen‐focused strategies, akin to the successful targeting of *A. baumannii* by SUL/DUR; and a renewed commitment to developing effective oral therapies to facilitate outpatient treatment and reduce healthcare burdens.

Ultimately, the preservation of *β*‐lactam antibiotics, the cornerstone of modern medicine, hinges on a sustained, global, and collaborative effort. The innovative molecules detailed in this review represent significant victories, but they are not a permanent solution. Their long‐term efficacy, and indeed the future of antimicrobial therapy, depends on integrating these pharmaceutical advances with robust global surveillance, unwavering investment in fundamental research, and the stringent implementation of antibiotic stewardship programs. The clinical utility of these life‐saving agents must be vigilantly guarded through collective action and prudent use, ensuring that this indispensable class of drugs remains effective for generations to come.

## Author Contributions

**Javad Yasbolaghi Sharahi:** conceptualization, supervision, writing – original draft, writing – review & editing. **Mohammad Javad Roustaye Gourabi:** investigation, writing – original draft, writing – review & editing. **Anita Nikoo:** investigation, writing – review & editing. **Bita Khanbabaei:** investigation, writing – review & editing. **Masoud Kargar:** investigation, visualization. **Amirhossein Fayyazi:** investigation, writing – review & editing. **Seyyed Mohammad Javad Mousavi:** investigation, writing – review & editing. **Amirhossein Aghdaee:** investigation, visualization. **Ali Hashemi:** conceptualization, writing – original draft.

## Funding

No funding was received for this manuscript.

## Disclosure

All authors read and approved the final manuscript.

## Consent

The authors have nothing to report.

## Conflicts of Interest

The authors declare no conflicts of interest.

## Data Availability

All data generated and analyzed during the current study are available from the corresponding author on reasonable request.
